# Reply to: Meta-analysis of vitamin D supplementation and hemoglobin concentration: methodological faults obscure the interpretation of the data

**DOI:** 10.1186/s12937-021-00682-9

**Published:** 2021-04-08

**Authors:** Seyyed Mostafa Arabi, Golnaz Ranjbar, Leila Sadat Bahrami, Abdolreza Norouzy

**Affiliations:** Metabolic Syndrome Research Center, Department of Nutrition, Faculty of Medicine, Mashhad University of Medical Sciences, Mashhad, 91179481564 Iran

Dear Editor,

We would like to thank Mr. Raeisi-Dehkordi, et al. [1] for their interest and knowledgeable comments on our study.

We have followed the PRISMA guidelines according to the most recent research standards (page 3 of the article). PICOS was also used in the study design (amendments are made on page 2). While it is not compulsory to submit all systematic reviews at PROSPERO, all the principles mentioned in PROSPERO were followed. In the corrected version of the paper [2], we have clarified that no restrictions were placed on the gender, race, and geographical distribution of the individuals enrolled in the study. However, age restrictions were places such that the studies carried out in subjects with a mean age of ≥17.5 years old were included. Also, the type of supplementation has been corrected to include all types of vitamin D supplementation and not just oral vitamin D supplementation. According to the Cochrane handbook, including multiple comparisons from one study with a shared intervention group to the meta-analysis may lead to bias [3]. However, in many published articles this method has been used. Therefore, we decided to use this method based on previous research [4, 5]. According to your suggestion, the stated combination of supplementary studies [6, 7] were removed from the final analysis and a new Forrest plot was introduced. However, no changes in the results were observed (Figs. 2, 3, 4 and 5).

**Fig. 2:**
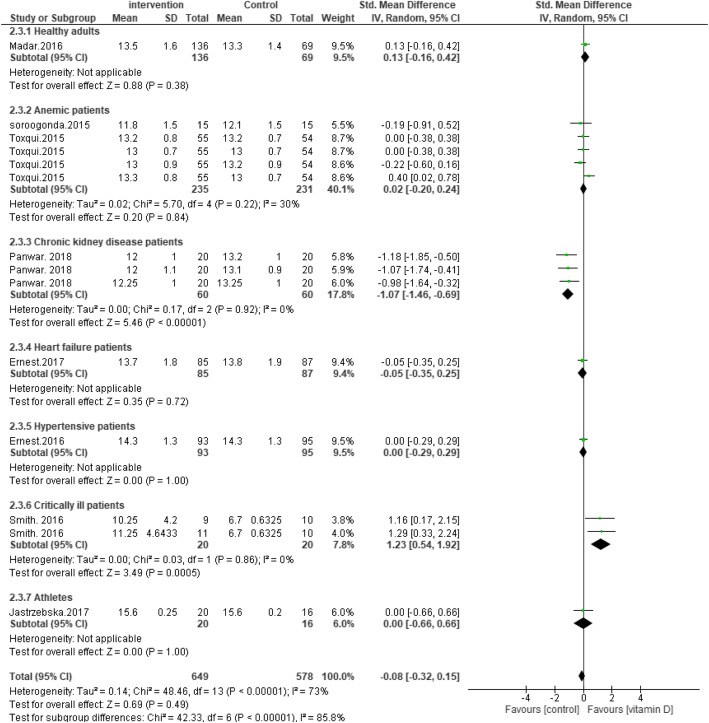
Forest plot showing results of a meta-analysis on the effects of vitamin D supplementation on hemoglobin. Data were reported as SMDs with 95% CIs

**Fig. 3:**
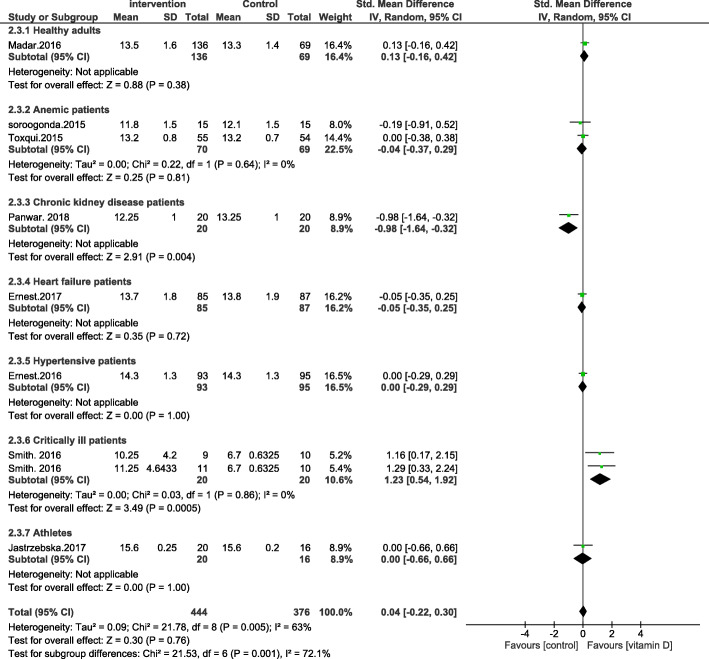
Forest plot showing results of a meta-analysis on the effects of vitamin D supplementation on ferritin. Data were reported as SMDs with 95% CIs

**Fig. 4:**
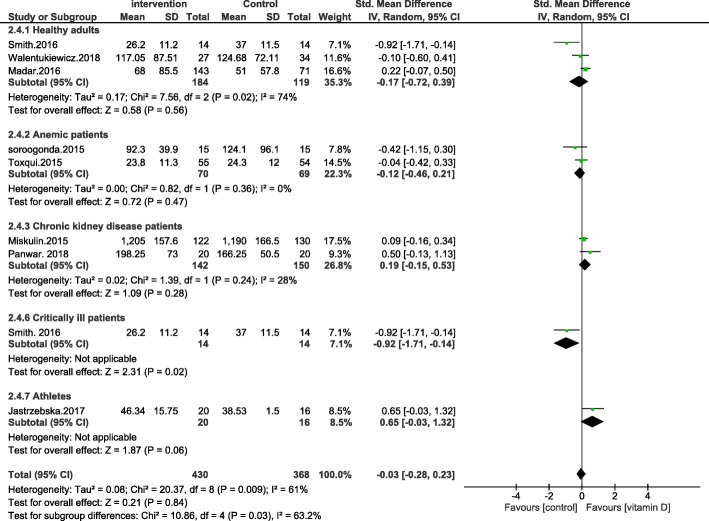
Forest plot showing results of a meta-analysis on the effects of vitamin D supplementation on transferrin saturation. Data were reported as MDs with 95% CIs

**Fig. 5:**
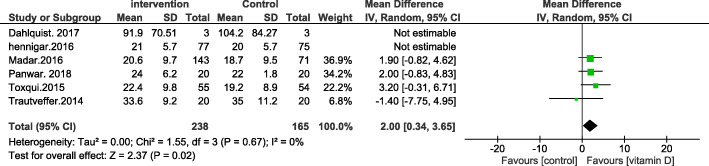
Forest plot showing results of a meta-analysis on the effects of vitamin D supplementation on iron levels. Data were reported as SMDs with 95% CIs

## References

[1] Zimorovat A, Mohammadi-Sartang M, Barati-Boldaji R, et al. Meta-analysis of vitamin D supplementation and hemoglobin concentration: methodological faults obscure the interpretation of the data. Nutr J. 2021;20:22. 10.1186/s12937-021-00681-w. 10.1186/s12937-021-00681-w PMC7980619 33740966

[2] Arabi SM, Ranjbar G, Bahrami LS, et al. Correction to: The effect of vitamin D supplementation on hemoglobin concentration: a systematic review and meta-analysis. Nutr J. 2021;20:21. 10.1186/s12937-021-00679-4. 10.1186/s12937-021-00679-4 PMC7934529 33663525

[3] Higgins JP, Thomas J, Chandler J, Cumpston M, Li T, Page MJ, et al. Cochrane handbook for systematic reviews of interventions: Wiley; 2019. 16.5.4 How to include multiple 173 groups from one study. Available from: https://handbook-5-174 1.cochrane.org/chapter_16/16_5_4_how_to_include_multiple_groups_from_one_study.htm 10.1002/14651858.ED000142 PMC10284251 31643080

[4] Jackson JK, Patterson AJ, MacDonald-Wicks LK, Oldmeadow C, McEvoy MA (2018). The role of inorganic nitrate and nitrite in cardiovascular disease risk factors: a systematic review and meta-analysis of human evidence. Nutr Rev.

[5] Mazidi M, Rezaie P, Vatanparast H (2018). Impact of vitamin D supplementation on C-reactive protein; a systematic review and meta-analysis of randomized controlled trials. BMC Nutr.

[6] Dahlquist DT, Stellingwerff T, Dieter BP, McKenzie DC, Koehle MS (2017). Effects of macro-and micronutrients on exercise-induced hepcidin response in highly trained endurance athletes. Appl Physiol, Nutr Metab.

[7] Hennigar SR, Gaffney-Stomberg E, Lutz LJ, Cable SJ, Pasiakos SM, Young AJ (2016). Consumption of a calcium and vitamin D-fortified food product does not affect iron status during initial military training: a randomised, double-blind, placebo-controlled trial. Br J Nutr.

